# Fatty Liver Index and mortality after myocardial infarction: A prospective analysis in the Alpha Omega Cohort

**DOI:** 10.1371/journal.pone.0287467

**Published:** 2023-09-08

**Authors:** Luc Heerkens, Laurens A. van Kleef, Robert J. de Knegt, Trudy Voortman, Johanna M. Geleijnse

**Affiliations:** 1 Division of Human Nutrition and Health, Wageningen University, Wageningen, The Netherlands; 2 Department of Gastroenterology and Hepatology, Erasmus MC, University Medical Centre Rotterdam, Rotterdam, The Netherlands; 3 Department of Epidemiology, Erasmus MC, University Medical Centre Rotterdam, Rotterdam, The Netherlands; Kurume University School of Medicine, JAPAN

## Abstract

Accumulating evidence shows that NAFLD might play a role in the etiology and progression of CVD, but little is known on the association of NAFLD and CVD mortality in patients with a history of a myocardial infarction (MI). Therefore, we studied the relationship of Fatty Liver Index (FLI), as indicator for non-alcoholic fatty liver disease (NAFLD), with 12-year risk of cardiovascular disease (CVD) and all-cause mortality in post-MI patients. We included 4165 Dutch patients from the Alpha Omega Cohort aged 60–80 years who had an MI ≤10 years prior to study enrolment. NAFLD was defined as FLI ≥60. Patients were followed for cause-specific mortality from enrolment (2002–2006) through December 2018. Hazard ratios for CVD and all-cause mortality were obtained by multivariable Cox regression using FLI <30 (indicating absence of NAFLD) as the reference. Baseline FLI as a continuous measure was studied with mortality using restricted cubic splines analyses. The median (IQR) FLI was 68 (48–84). Sixty percent of the patients had FLI ≥60, who were more likely to be male and more often had diabetes, high blood pressure, and high serum cholesterol levels. During 12 years of follow-up, 2042 deaths occurred of which 846 from CVD. Patients with NAFLD were at increased risk of CVD mortality (HR: 1.55 [1.19, 2.03]) and all-cause mortality (HR: 1.21 [1.03; 1.41]) compared to patients without NAFLD. Results remained consistent after excluding patients with obesity and diabetes. To conclude, the adverse association of FLI with CVD mortality was stronger in female than in male patients with conventional cut-off points. FLI ≥60, indicating NAFLD, was a predictor for CVD and all-cause mortality in post-MI patients, independent of other cardiometabolic risk factors. However, cut-off points might differ between male and female patients for predicting CVD mortality.

## Introduction

Cardiovascular disease (CVD) is the leading cause of death worldwide, of which myocardial infarction (MI) is one of the predominant causes [1]. Mortality rates for CVD have declined over the past decades in Western countries. However, the number of patients with prevalent CVD is increasing, which may be attributed to the ageing population and better survival because of improved treatment, along with increasing prevalences of metabolic disorders, such as obesity and type 2 diabetes [1].

Non-alcoholic fatty liver disease (NAFLD) has been regarded as the hepatic manifestation of the metabolic syndrome. NAFLD comprise a broad spectrum ranging from simple steatosis to steatohepatitis, fibrosis, and cirrhosis, not caused by heavy alcohol consumption [2] and is a dynamic condition which can progress and regress over time [3,4]. Accumulating evidence shows that NAFLD might play a role in the etiology and progression of CVD [5]. However, whether NAFLD contributes to CVD independently of type 2 diabetes and obesity remains unclear, which is especially important since NAFLD is closely associated with cardiometabolic risk factors [6,7].

The Fatty Liver Index (FLI) is a predictor of the presence of NAFLD [8,9]. Its use is endorsed in epidemiological studies, because of its non-invasiveness and simplicity [10]. The FLI, based on BMI, waist circumference, triglycerides and the liver enzyme gamma-glutamyl transferase (GGT) has been validated for the presence of NAFLD by ultrasonography in a population-based cohort [8].

Little is known on the association of NAFLD and CVD mortality in post-MI patients. Associations in this group may differ due to cardiometabolic alterations, medication use, and older age. Only one prospective cohort study has been performed in CVD patients, showing a higher CVD mortality risk with higher FLI in >3200 persons who were referred to the hospital for coronary angiography (of which 40% had an MI) [11]. Therefore, we examined whether FLI, as an indicator of NAFLD, could predict mortality in the Alpha Omega Cohort, a large prospective cohort study of older Dutch post-MI patients using state-of-the-art medication. Associations were obtained for FLI assessed at baseline and for 40-month changes in FLI, in relevant subgroups by sex, age, and underlying CVD risk.

## Methods

### Study design and patients

The Alpha Omega Cohort originates from an intervention study of low-dose omega-3 fatty (Alpha Omega Trial), which showed no effect on major cardiovascular events [12,13]. After the trial, the Alpha Omega Cohort continued as a prospective cohort study and patients have been continuously monitored for cause-specific mortality. The study was approved by a central ethics committee (Haga Hospital) and by the ethics committees of all participating hospitals. Patients signed written informed consent.

At baseline (2002–2006), the Alpha Omega Cohort comprised 4837 patients who experienced a myocardial infarction ≤10 years prior to their study enrolment. Patients missing data on FLI components (n = 378) were excluded. Patients with missing data on alcohol consumption (n = 200) or excessive alcohol consumption (>30 grams/day for men or >20 grams/day for women, n = 344) were excluded as well, resulting in a population of 4165 patients for studying baseline FLI and mortality risk (S1 Fig). Measurements were repeated in a random subset of the cohort which was re-examined after approximately 40 months (n = 2503) After applying the same exclusion criteria, 1678 patients were available for the analysis of 40-month change in FLI and mortality risk.

### Fatty liver index

FLI was calculated at baseline and after 40 months of follow-up on the basis of BMI (kg/m^2^), waist circumference (cm), fasted and non-fasted serum GGT (U/L), and fasted or non-fasted serum triglycerides (mg/dL). Physical examination was performed in the hospital or at the patients’ homes by trained research nurses. Weight and height were measured, and BMI was calculated as body weight divided by square height (kg/m^2^). Waist circumference (cm) was measured at the midpoint between the bottom rib and the top of the hipbone using a non-elastic tape. Venous blood samples (30 mL) were drawn fasted (>8 hours, 35% of the population for analysis) or non-fasted and were sent to the laboratory by next-working-day mail service. Upon arrival, blood samples were immediately processed and stored at -80 degrees Celsius. Serum triglycerides were determined by standard assays (Roche Diagnostics; cat. no. 1488872) on an automated analyzer (Hitachi 912; Roche Diagnostics) with an inter- and intra-assay coefficient of variation <10%. Fasted and non-fasted serum GGT was determined by standard assays (Abbott Diagnostics; cat. no. 7D6522) on an automated analyzer (ARCHITECT ci8200; Abbott) with an intra- and inter-assay coefficient of variation <10%.

We used the conventional cut-off points of FLI <30 to indicate the absence of hepatic steatosis and FLI ≥60 to indicate the presence of hepatic steatosis. These cut-off points have been validated in a Caucasian population-based cohort showing a probability of 87% for absence and 86% for presence of NAFLD [8], and is calculated as follows:

$$FLI=(e^{0.953*loge(triglycerides)+0.139*BMI+0.718*loge(GGT)+0.053*waist\;circumference‐15.745})/(1+e^{0.953*loge(triglycerides)+0.139*BMI+0.718*loge(GGT)+0.053*waist\;circumference‐15.745})\;*\;100$$

### Mortality endpoints

The primary endpoints of the current study were CVD mortality and all-cause mortality. Patients were monitored for their vital status from baseline through 31 December 2018 through linkage with municipal registries. Data collection on cause-specific mortality occurred in three phases. During the Alpha Omega Trial (2002–2009), information was obtained from the national mortality registries [Statistics Netherlands (CBS)], treating physicians, and close family members. Primary and contributing causes of death were coded by an independent Endpoint Adjudication Committee and described in detail elsewhere [12,13]. After the trial through 2012, mortality data were obtained from CBS for primary and contributing causes of death. From 2013 onwards, data on only primary cause of death were obtained from CBS. Treating physicians filled out an additional cause-of-death questionnaire (response rate: 67%), which was coded by study physicians who were not involved in the current analysis. The endpoint CVD was allocated to all patients for whom it was a primary or contributing cause of death, based on any of the data sources.

Fatal endpoints were coded according to the International Classification of Diseases, 10^th^ revision (ICD-10) [14]. CVD mortality comprised coronary heart diseases (codes I20-I25), cardiac arrest (I46), heart failure (I50), stroke (I60-I69), and undefined sudden death (R96).

### Other measurements

Data at baseline and after 40 months of follow-up were collected on demographics, smoking status, alcohol consumption, and medication use.

Self-reported smoking status was assessed in four categories (current, quit ≤10 years, quit >10 years, never). Self-reported medication use was checked by research nurses and coded according to the Anatomical Therapeutic Chemical Classification System (ATC). The ATC code for statins was C10A, A10 for antidiabetic medication, and C02, C03, C07, C08, and C09 for antihypertensives. The intake of alcoholic beverages (frequency, amount) over the past month was assessed using a Food Frequency Questionnaire [15], from which total ethanol intake (g/d) was computed. Alcohol consumption in this population with non-excessive alcohol intake (< 20 g/d in female or < 30 g/d in male) was categorized as follows: abstinence (0 g/d), light (>0–10 g/d), and moderate (>10 g/d) [16]. Trained research nurses measured blood pressure at baseline and after 40 months of follow-up. Blood pressure was measured twice on each occasion after a 15-minute rest, and values were averaged.

Serum alanine aminotransferase (ALT) and aspartate aminotransferase (AST) were analyzed in stored blood by standard assays (Abbott Diagnostics; cat. no. 8L9222 and 8L9122) on an automated analyzer (ARCHITECT ci8200; Abbott) with an intra- and inter-assay coefficient of variation <10%. Prevalent diabetes was assessed by physician’s diagnosis, use of diabetic medication or when the patient had elevated plasma glucose (≥7.0 mmol/L if fasted >4 h or ≥11.0 mmol/L if not fasted.

### Statistical analysis

Baseline characteristics are presented across categories of FLI (<30; ≥30-<60, ≥60) as means ± SD for normally distributed data, medians and interquartile range (IQR) for skewed data, and *n* (%) for categorical data.

Cox proportional hazards models were used to estimate hazard ratios (HRs) with 95% confidence intervals (95% CIs) for CVD mortality, CHD mortality, and all-cause mortality. In the main models, FLI at baseline was analyzed in categories, using FLI <30 as the reference category. The assumption of proportionality was checked visually using a log-minus-log plot of survival and time and was met. Survival time (in person-years) was calculated from the date of enrolment to date of death or end of follow-up. One person was lost to follow-up and censored after 2.9 years.

Unadjusted HRs were presented in model 1. Subsequently, HRs were adjusted for age and sex (model 2). Model 3 also included systolic blood pressure (mmHg), smoking status (4 categories), alcohol consumption (3 categories), statin use (yes/no), time since last MI (years), fasting, and the type of intervention during the Alpha Omega Trial (types of omega-3 fatty acids or placebo; 4 categories). The latter, however, was not a confounder because it was randomly assigned. Missing data for smoking status (n = 1), systolic blood pressure (n = 6), and time since last MI (n = 41) were imputed with a sex-specific mode, mean or median, respectively.

Restricted cubic splines (RCS) analyses were performed to assess the continuous association of FLI with CVD and all-cause mortality, using the fully adjusted model. FLI = 30 was set as the reference and four knots were placed at the 5^th^, 35^th^, 65^th^, and 95^th^ percentiles. The number of these knots were chosen according to the lowest Akaike’s Information Criteria [17]. The Wald chi-square test was performed to test for nonlinearity [18]. RCS analyses were repeated and stratified by sex to assess effect measure modification.

We performed a series of additional analyses. We stratified the main analysis, studying FLI categories, for sex and age. Furthermore, we repeated the main analysis in non-obese (n = 3163) and non-diabetic (n = 3308) patients to study FLI ≥60 as a predictor of CVD and all-cause mortality independent of obesity and diabetes. Analyses were performed for single FLI components (expressed as Z-scores) and AST/ALT ratio, as an additional diagnostic marker for liver diseases [19], in relation to CVD and all-cause mortality. Furthermore, we repeated the main analysis using FLI <60 as the reference category. Sensitivity analyses were performed by excluding the first two years of follow-up and repeating the main analysis using the first 10 years of follow-up.

In our cohort of patients who underwent an assessment 40 months after baseline, we repeated RCS analyses to study the change of FLI after 40 months of follow-up (baseline FLI–FLI after 40 months of follow-up; expressed as Z-scores) in relation to CVD and all-cause mortality, excluding the first 40 months of follow-up. Zero change of FLI was set as the reference and the analyses were additionally stratified for NAFLD at baseline (FLI <60; and FLI ≥60).

Two-sided P-values <0.05 were considered statistically significant. Statistical analyses were performed using statistical software SAS version 9.4 (SAS Institute Inc.) and R version 4.1.0 (R Foundation for Statistical Computing).

## Results

Patients had a mean ± SD age of 69.1 ± 5.7 years, 23% was female, 85% used statins. Patients had a mean BMI of 27.8 ± 3.9 and a median FLI of 68 (48–84). Table 1 presents baseline characteristics of these patients (n = 4165), overall and across FLI categories (<30, 30–60, ≥60). Sixty percent had an FLI ≥60, indicating NAFLD and those were more likely to be male and had more often diabetes, higher blood pressure and higher serum cholesterol. During a median (IQR) follow-up of 12 years (8.5–14.0) (total of 46,072 person-years) we observed 1934 deaths (incidence rate: 42 per 1000 person-years), including 795 deaths from CVD (17 per 1000 person-years) and 593 deaths from CHD (13 per 1000 person-years).

**Table 1:** Baseline characteristics of 4165 post-MI patients of the Alpha Omega Cohort, overall and across categories of FLI^a^.

		Fatty Liver Index		
	Total (n = 4165)	<30 (n = 384)	≥30-<60 (n = 1262)	≥60 (n = 2519)
FLI	68 (48–84)	21 (16–25)	48 (39–54)	81 (70–90)
Fasting FLI^b^	63 (45–80)	20 (15–25)	48 (40–53)	78 (69–89
Male	3347 (77.0)	251 (62.3)	1012 (77.3)	2084 (79.1)
Age	69.1 ± 5.7	69.8 ± 5.8	69.5 ± 5.3	68.8 ± 5.6
BMI, kg/m^2^	27.8 ± 3.9	22.7 ± 1.9	25.6 ± 2.0	29.6 ± 3.5
Waist circumference, cm	102 ± 11	86 ± 7	96 ± 6	107 ± 9
Smoking status^c^				
Never	773 (17.8)	90 (22.3)	274 (20.9)	409 (15.5)
Former, >10 years ago	761 (17.5)	64 (15.9)	245 (18.7)	452 (17.2)
Former, ≤10 years ago	2103 (48.4)	164 (40.7)	587 (44.8)	1352 (51.3)
Current	709 (16.3)	85 (21.1)	204 (15.6)	420 (16.0)
Alcohol consumption^d^				
Abstinence	572 (13.7)	60 (15.6)	156 (12.4)	356 (14.1)
Light	2665 (64.0)	258 (67.2)	813 (64.4)	1594 (63.3)
Moderate	928 (22.3)	66 (17.2)	293 (23.3)	569 (22.6)
Time since last MI^e^, years	3.7 (1.7–6.3)	2.8 (1.2–5.7)	3.2 (1.5–5.9)	4.0 (1.9–6.6)
Hypertension^f^	4139 (95.2)	371 (92.1)	1231 (94.0)	2537 (96.3)
Hypercholesterolaemia^g^	4171 (96.0)	378 (93.8)	1252 (95.6)	2541 (96.5)
Diabetes	902 (20.7)	55 (13.6)	171 (13.1)	676 (25.7)
Plasma glucose^h^, mmol/L	6.21 (2.09)	5.52 ± 1.54	5.72 ± 1.41	6.56 ± 2.36
Serum lipids, mmol/L				
LDL cholesterol^i^	2.57 ± 0.83	2.50 ± 0.83	2.58 ± 0.80	2.58 ± 0.85
HDL cholesterol	1.28 ± 0.34	1.52 ± 0.40	1.36 ± 0.34	1.20 ± 0.30
Triglycerides	1.65 (1.21–2.31)	1.05 (0.86–1.28)	1.34 (1.07–1.69)	2.00 (1.50–2.72)
Fasting triglycerides^b^	1.45 (1.10–1.94)	1.02 (0.83–1.24)	1.22 (1.00–1.51)	1.77 (1.37–2.31)
Total cholesterol	4.70 ± 0.97	4.53 ± 0.97	4.59 ± 0.92	4.79 ± 0.98
Liver enzymes, U/L				
Gamma-glutamyltransferase	32 (24–47)	21 (17–27)	27 (21–36)	38 (28–56)
Fasting gamma-glutamyltransferase^b^	32 (24–46)	22 (18–27)	28 (22–37)	39 (29–55)
Alanine transaminase^j^	16 (13–22)	13 (10–17)	15 (12–20)	18 (14–24)
Aspartate transaminase^k^	27 (24–32)	26 (23–31)	27 (24–31)	28 (24–33)
AST/ALT ratio^l^	1.8 ± 0.9	2.1 ± 0.6	1.9 ± 1.1	1.7 ± 0.8
Systolic blood pressure^m^, mmHg	144 ± 22	138 ± 23	141 ± 21	142 ± 21
Diastolic blood pressure^m^, mmHg	80 ± 11	78 ± 11	80 ± 11	81 ± 11
Medication use				
Statins	3712 (85.4)	327 (81.1)	1137 (86.8)	2248 (85.3)
Antihypertensive drugs	3901 (89.7)	347 (86.1)	1144 (87.3)	2410 (91.5)
Antidiabetic drugs^n^	661 (15.2)	38 (9.4)	122 (9.3)	501 (19.0)

^a^ Values are means ± SDs for normally distributed variables, medians (IQRs) for skewed variables, or n (%) for categorical variables. FLI, Fatty Liver Index.

^b^ Fasting for >8 hours (n = 1458).

^c^ Missing data for 1 patients.

^d^ Abstinence, 0 g/d; light, >0–10 g/d; moderate, >10 g/d

^e^ Missing data for 41 patients.

^f^ Defined as use of antihypertensives or newly diagnosed from enrolment.

^g^ Defined as use lipid-lowering medication or newly diagnosed from enrolment.

^h^ Missing data for 17 patients.

^i^ Missing data for 198 patients.

^j^ Missing data for 51 patients.

^k^ Missing data for 1 patient.

^l^ Missing data for 52 patients.

^m^ Missing data for 6 patients.

^n^ Oral medication and insulin (analogues).

Table 2 shows the HRs (95% CI) in FLI categories in relation to CVD and all-cause mortality risk. In model 1, FLI ≥60 compared to <30 was associated with a higher risk of CVD mortality (model 1: HR: 1.34; 95% CI: 1.02, 1.75), which was higher after adjustment for potential confounders (model 3: HR: 1.58; 95% CI: 1.19, 2.09). For all-cause mortality, a higher risk with FLI ≥60 was found as well, compared to FLI <30 (model 3: HR: 1.18; 95% CI: 1.00, 1.39).

**Table 2:** HRs (95% CIs) for CVD mortality and all-cause mortality in categories of FLI in 4165 post-MI patients of the Alpha Omega Cohort.

	Fatty Liver Index		
	<30 (n = 384)	≥30-<60 (n = 1262)	≥60 (n = 2519)
CVD mortality			
Cases	58	226	511
Person-years	4392	14,819	28,644
Incidence rate (per 1000 person-years)	13.2	15.2	17.8
Model 1	1.00	1.12 (0.84; 1.49)	1.34 (1.02; 1.75)
Model 2	1.00	1.16 (0.87; 1.54)	1.50 (1.14; 1.97)
Model 3	1.00	1.27 (0.95; 1.71)	1.58 (1.19; 2.09)
All-cause mortality			
Cases	172	557	1205
Person-years	4392	14,819	28,644
Incidence rate (per 1000 person-years)	39.1	37.5	42.0
Model 1	1.00	0.93 (0.78; 1.10)	1.06 (0.91; 1.25)
Model 2	1.00	0.94 (0.79; 1.12)	1.16 (0.99; 1.36)
Model 3	1.00	1.01 (0.85; 1.20)	1.18 (1.01; 1.39)

Hazard ratio (95% confidence interval) obtained from Cox proportional hazards models, using the lowest category as the reference. CVD, cardiovascular diseases; CHD coronary heart diseases; FLI, Fatty Liver Index.

Model 2 adjusted for sex and age.

Model 3, as model 2 and additionally adjusted for systolic blood pressure, statin use, smoking status, alcohol consumption, time since last myocardial infarction, and fasting.

**Fig 1:**
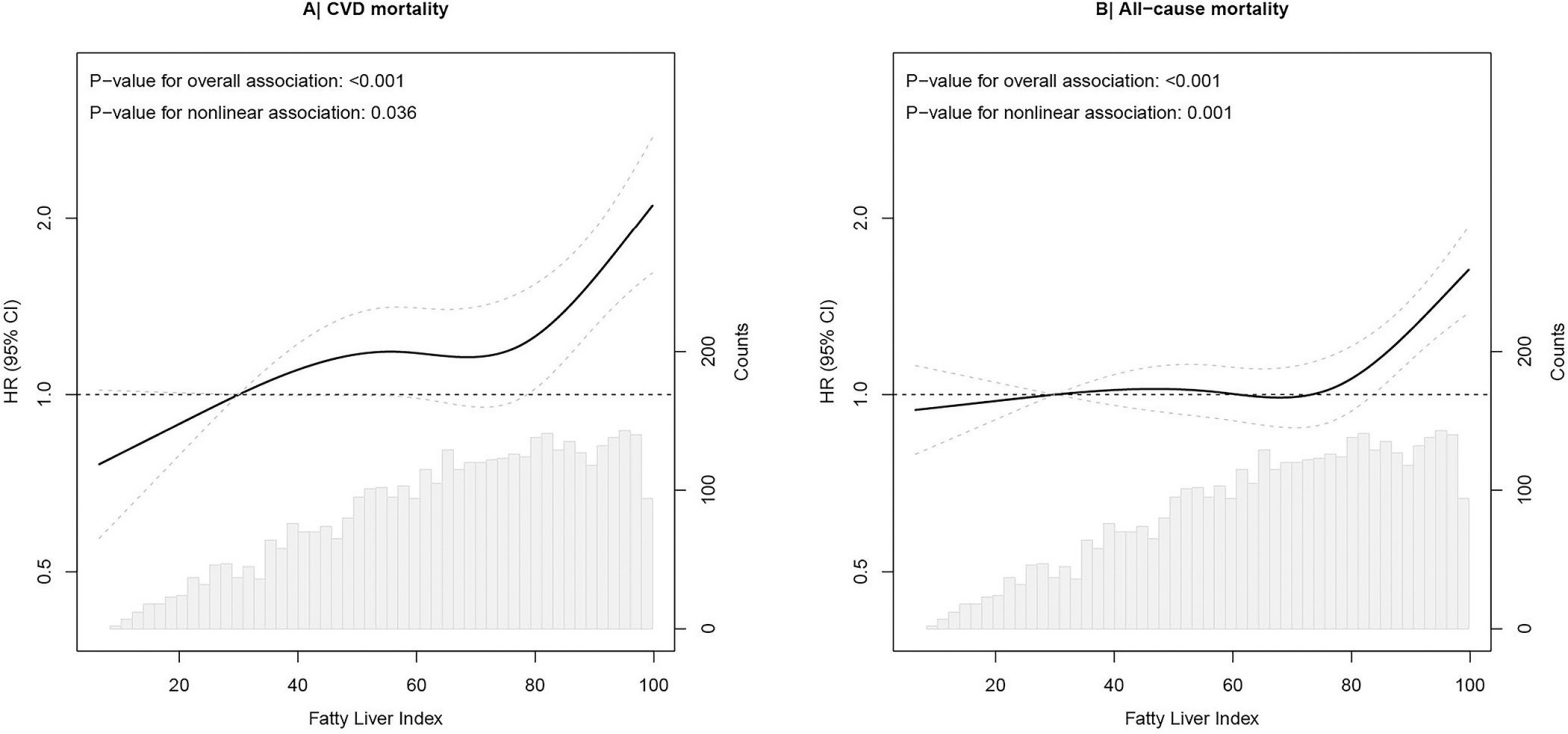
Associations in hazard ratios of FLI as continuous exposure in relation to CVD mortality (A) and all-cause mortality (B) in 4165 post-MI patients of the Alpha Omega Cohort. Hazard ratios with 95% CIs (dotted line) were modeled using restricted cubic splines. Four knots for FLI are located at the 5th, 35th, 65th, and 95th percentiles. Hazard ratios are adjusted for age, sex, systolic blood pressure, smoking status, alcohol consumption, time since last myocardial infarction, and fasting. CVD, cardiovascular diseases; FLI, Fatty Liver Index.

RCS analyses for FLI and primary mortality endpoints are shown in Fig 1. Using FLI = 30 as the reference, FLI was nonlinearly associated with CVD mortality with a lower risk for FLI <30 and a gradual increase from FLI >75 (Fig 1). FLI between >30 and 75 was not associated with CVD mortality, compared to FLI = 30. Results for all-cause mortality showed no association with FLI up to 75. With FLI >75 we observed a slight increase in mortality risk.

**Fig 2:**
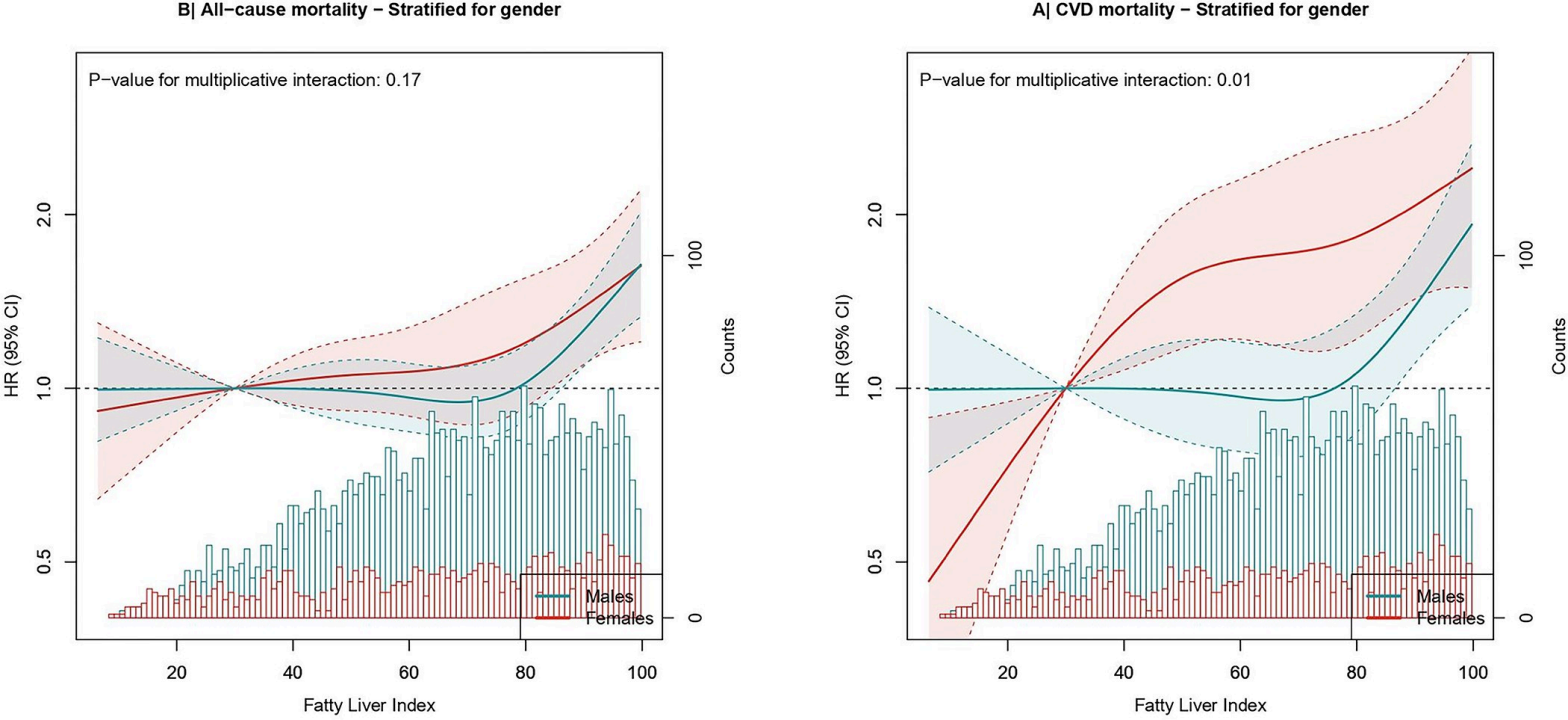
Stratified associations by sex in hazard ratios of FLI as continuous exposure in relation to CVD mortality (A) and all-cause mortality (B) in 4165 post-MI patients from the Alpha Omega Cohort. Hazard ratios with 95% CIs (dotted line) were modeled using restricted cubic splines. Four knots for FLI are located at the 5th, 35th, 65th, and 95th percentiles. Hazard ratios are adjusted for age, sex, systolic blood pressure, smoking status, alcohol consumption, time since last myocardial infarction, and fasting. CVD, cardiovascular diseases; FLI, Fatty Liver Index.

RCS analyses stratified by sex are presented in Fig 2. FLI <30 was associated with a lower risk and FLI >30 with a gradually increased risk of CVD mortality among female patients, compared to FLI = 30 (Fig 2). Among male patients, FLI <75 was not associated with CVD mortality, compared to FLI = 30. FLI ≥75 was associated with a higher CVD mortality risk among male patients (Fig 2). Multivariable HRs differed substantially between males and females for categorical analyses (S2 Fig), showing an increased risk of CVD mortality for females with an FLI ≥60 compared to FLI <30 (HR of 2.66 (1.55, 4.56). In men, FLI ≥60 was associated with an increased risk of CVD mortality, which was not statistically significant (HR of 1.13 (0.82; 1.56)). Because the FLI was skewed and predicted CVD mortality at different levels for male and female patients, we decided to analyze the FLI in tertiles (<50; 50–74; ≥75). Male and female patients with FLI ≥75 had a higher risk of CVD HR of 1.27 (1.03, 1.58) and (HR of 2.08 (1.44, 2.99), respectively), compared to FLI <50 (S3 Fig). HRs for all-cause mortality analyzing FLI with conventional cut-off points, in tertiles, and as continuous measure did not differ substantially between male and female patients.

HRs for CVD mortality were slightly lower in patients ≤70 years (HRs of 1.30 (0.83, 2.05)) than in patients aged >70 years with FLI ≥60 (HR of 1.48 (1.07, 2.05); S2 Fig). HRs did not change when excluding patients with obesity (HR of 1.50 (1.13, 1.99); S1 Table) or diabetes (HR of 1.54 (1.12, 2.13); S2 Table). Individual FLI components were all positively associated with risk of CVD mortality of which GGT (log transformed due to skewness) was associated with the highest mortality risk (HR of 1.20 per Z-scores (1.15, 1.26); S3 Table). We also observed a 5% increased risk of CVD mortality per one SD higher AST/ALT ratio (model 3: HR: 1.04; 95% CI: 1.00, 1.09). Results for all-cause mortality were similar to those for CVD mortality for all FLI components. HRs were attenuated, but remained statistically significant when FLI <60 was used as the reference (S4 Table). Excluding the first two years of follow-up (total of 37,877 person-years; S5 Table) and using the first 10 years of follow-up (total of 18,573 person-years; S6 Table) yielded similar results as for the main analysis, although they were no longer statistically significant.

In 1678 patients with repeated measurements, we observed a mean absolute change in FLI of 10.5 (± 9.3) after 40 months of follow-up (S7 Table). The largest change of FLI components was with GGT (15.1 ± 41.8). Of 1678 these patients, 142 progressed into FLI ≥60 after 40 months of follow-up, while 177 patients regressed from FLI ≥60. Change of FLI was not associated with CVD mortality, compared to the reference (0 change of FLI) using RCS analyses (S4 Fig). After stratification of baseline FLI (FLI <60; FLI ≥60), a decrease in FLI tended to be associated with a lower risk of CVD mortality for patients with a baseline FLI ≥60, but not in those with baseline FLI <60. Change of FLI was not associated with all-cause mortality risk in the total study population, but an increase of FLI was associated with a higher all-cause mortality risk for patients with a baseline FLI ≥60 (S5 Fig).

## Discussion

We showed that 60% of 4165 Dutch post-MI patients aged 60–80 years had FLI ≥60, suggesting the presence of NAFLD. FLI ≥60 was associated with a 58% higher risk of CVD mortality and a 18% higher risk of all-cause mortality after a median follow-up of 12 years, compared to FLI <30. When we analyzed FLI as a continuous variable using non-linear models we observed that for male patients, FLI ≥75 was associated with a higher CVD mortality risk. For female patients FLI >30 was associated with a higher CVD mortality risk. For all-cause mortality, findings were similar both in male and female patients.

Studies on NAFLD and mortality after MI are scarce. Our results are in line with a study of 3207 German patients who were referred to the hospital for coronary angiography. After a median follow-up of 7.7 years, an HR of 1.64 (95% CI: 1.07, 2.51) for CVD mortality and 1.26 (95% CI: 1.02, 1.54) for all-cause mortality with the highest quartile (FLI >75.6) compared to the lowest quartile was observed [11]. We observed similar effect estimates, in both categorical analyses using clinical FLI cutoffs and in non-linear analyses in which we observed a higher CVD and all-cause mortality risk starting at FLI of 75.

HRs for FLI and CVD mortality differed between male and female post-MI patients of the Alpha Omega Cohort. Only few studies have examined the association of NAFLD with mortality risk stratified for gender. A meta-analysis of Liu et al. [20] with studies in population-based and diabetic patients cohorts addressed the sex difference of the association of NAFLD with CVD mortality. Only two of the included cohorts stratified for sex and showed a pooled HR of 0.93 (0.67, 1.29) for males and 1.25 (0.76, 2.06) for females with NAFLD as compared to no NAFLD, assessed with ultrasonography [20]. In our Alpha Omega Cohort, a lower risk was associated with FLI <30 and a gradual increase in mortality risk with FLI >30 was observed in female patients. In male patients, however, the risk of CVD mortality did not differ for FLIs across the wide range of <30 through 75. Only with FLI ≥75 a higher mortality risk was found which was similar to the analyses using tertiles. However, HRs for FLI in tertiles still differed between male and female patients. Although FLI ≥60 indicates NAFLD, our results may advocate to use different cut-off points for predicting mortality in post-MI patients, which may need to be sex-specific for CVD mortality.

Biological mechanisms explaining through which hepatic lipid accumulation contributes to the progression of CVD is by promoting atherogenic dyslipidemia [21], but is unlikely due to the use of lipid-lowering medication in the post-MI patients of the Alpha Omega Cohort. Other possible mechanisms are the systemic release of inflammatory factors and insulin resistance as a consequence of hepatic steatosis, of which the latter is also a precursor that amplifies hepatic lipid accumulation and vice versa [21]. Since NAFLD is strongly related with diabetes and also obesity it can be questioned whether the association of FLI with CVD mortality risk is driven by other cardiometabolic risk factors. When we excluded obese and diabetic post-MI patients HRs only slightly attenuated, which suggested that the associations of FLI with CVD mortality risk were independent from obesity and diabetes.

Furthermore, we observed that the FLI remained approximately stable during a 40-month period in our cohort of 60–80 year-old post-MI patients, which can probably be explained by the relatively short follow-up or the older age of the cohort. With ageing it becomes more unlikely to change (lifestyle) habits, such as weight reduction. Weight is one of the largest predictors for NAFLD [4], but changes in BMI were small in the Alpha Omega Cohort. Only few patients progressed into FLI ≥60 or regressed from FLI ≥60 and, therefore, we were unable to study regression or progression of FLI ≥60 in relation to mortality. Moreover, changes in FLI were not statistically significantly associated with mortality in our cohort with post-MI patients.

Our study in this unique cohort of post-MI patients has limitations. First, although the sample size was large, only 384 patients (~10% of the cohort) had FLI <30 and represented the reference category which probably has resulted in wider CIs. To overcome this problem, we studied the FLI as continuous measure as well. Also, when FLI <60 was used as a reference category HRs slightly attenuated, but were still statistically significant. Second, only 35% of our cohort was fasted at time of blood collection, which could have affected measurements of blood parameters, such as serum triglycerides and GGT. However, adjusting for fasting status did not change the HRs. The strength of the current study is the long follow-up of >10 years for cause-specific mortality and only 1 patient was lost to follow-up.

Future research is warranted to explain the large differences for FLI and CVD mortality between male and female patients and to what extent the FLI and its cut-off points reflect NAFLD in male and female post-MI patients using more sophisticated methods to diagnose NAFLD, such as ultrasonography. Although the FLI is a non-invasive and validated marker for both males and females, it merely predicts the presence of NAFLD [8,9]. The likelihood of comorbidities or deteriorating health (e.g. presence of cancer, heart failure or COPD) in this patient population could cause a decline in BMI, thereby lowering FLI. It raises the question whether a low FLI resembles a healthy liver and this could have biased our reference category. Furthermore, we were not able to further define NAFLD severity, which has been associated with a gradual increase in mortality risk in a meta-analysis with population-based cohorts [5]. We did study the AST/ALT ratio, as marker for hepatic fibrosis, in relation to mortality, showing a positive association. More research is necessary with more sophisticated measures to study different stages of NAFLD with mortality. Furthermore, we were unable to study whether progression or regression of NAFLD is associated with mortality in post-MI patients and, therefore, needs clarification too.

In conclusion, a large proportion of post-MI patients was most likely to suffer from NAFLD, estimated on the basis of FLI. FLI was highly predictive for premature CVD mortality and all-cause mortality, but FLI cut-off points might differ between males and females regarding CVD mortality. Further studies are warranted to investigate if FLI cut-off points truly predict NAFLD, taking into account the likelihood of deteriorating health in this post-MI patient population.

## Supporting information

S1 Fig Flowchart for selection of the population for analysis in the Alpha Omega Cohort. (TIF)

S2 Fig Hazard ratios (95% confidence interval) for FLI in relation to CVD mortality (A) and all-cause mortality (B), stratified for sex and age (≤70 y and >70 y) in 4165 post-MI patients of the Alpha Omega Cohort. Hazard ratios are adjusted for age (when stratified for sex), sex (when stratified for age), systolic blood pressure, smoking status, alcohol consumption, time since last myocardial infarction, and fasting. CVD, cardiovascular diseases; FLI, Fatty Liver Index; HR, Hazard ratio; 95% CI, 95% confidence interval; IR, Incidence rate (per 1000 person-years).
(TIF)

S3 Fig Hazard ratios (95% confidence interval) for tertiles of FLI in relation to CVD mortality (A) and all-cause mortality (B), stratified for sex in 4165 post-MI patients of the Alpha Omega Cohort. Hazard ratios are adjusted for age, systolic blood pressure, smoking status, alcohol consumption, time since last myocardial infarction, and fasting. CVD, cardiovascular diseases; FLI, Fatty Liver Index; HR, Hazard ratio; 95% CI, 95% confidence interval; IR, Incidence rate (per 1000 person-years).
(TIF)

S4 Fig Associations in hazard ratios of change of FLI (expressed in Z-scores) in relation to CVD mortality in 1678 post-MI patients of the Alpha Omega Cohort, overall (A), for patients with baseline FLI <60 (B), and for patients with baseline FLI ≥60 (C). Hazard ratios with 95% CIs (dotted line) were modeled using restricted cubic splines. Three knots for FLI are located at the 10th, 50th, and 90th percentiles. Hazard ratios are adjusted for age, sex, systolic blood pressure, smoking status, alcohol consumption, time since last myocardial infarction, and fasting. CVD, cardiovascular diseases; FLI, Fatty Liver Index.
(TIF)

S5 Fig Associations in hazard ratios of change of FLI (expressed in Z-scores) in relation to CVD mortality in 1678 post-MI patients of the Alpha Omega Cohort, overall (A), for patients with baseline FLI <60 (B), and for patients with baseline FLI ≥60 (C). Hazard ratios with 95% CIs (dotted line) were modeled using restricted cubic splines. Three knots for FLI are located at the 10th, 50th, and 90th percentiles. Hazard ratios are adjusted for age, sex, systolic blood pressure, smoking status, alcohol consumption, time since last myocardial infarction, and fasting. FLI, Fatty Liver Index.
(TIF)

S1 Table HRs (95% CIs) for FLI in relation to CVD mortality and all-cause mortality in 3163 non-obese post-MI patients of the Alpha Omega Cohort. Hazard ratio (95% confidence interval) obtained from Cox proportional hazards models, using the lowest category as the reference. CVD, cardiovascular diseases; FLI, Fatty Liver Index. Model 2 adjusted for sex and age. Model 3, as model 2 and additionally adjusted for systolic blood pressure, statin use, smoking status, alcohol consumption, time since last myocardial infarction, and fasting.
(DOCX)

S2 Table HRs (95% CIs) for FLI in relation to CVD mortality and all-cause mortality in 3308 non-diabetic post-MI patients of the Alpha Omega Cohort. Hazard ratio (95% confidence interval) obtained from Cox proportional hazards models, using the lowest category as the reference. CVD, cardiovascular diseases; FLI, Fatty Liver Index. Model 2 adjusted for sex and age. Model 3, as model 2 and additionally adjusted for systolic blood pressure, statin use, smoking status, alcohol consumption, time since last myocardial infarction, and fasting.
(DOCX)

S3 Table HRs (95% CIs) for single FLI components and AST/ALT ratio in Z-scores in relation to CVD mortality and all-cause mortality in 4165 post-MI patients of the Alpha Omega Cohort. Hazard ratio (95% confidence interval) obtained from Cox proportional hazards models, using the lowest category as the reference. CVD, cardiovascular diseases; FLI, Fatty Liver Index. Model 2 adjusted for sex and age. Model 3, as model 2 and additionally adjusted for systolic blood pressure, statin use, smoking status, alcohol consumption, time since last myocardial infarction, and fasting. ^a^ Log transformed due to skewness of the data. ^b^ Fasting and non-fasting triglycerides. ^c^ Missing data for 48 patients (n = 4117).
(DOCX)

S4 Table HRs (95% CIs) for FLI ≥60 compared to FLI <60 at baseline in relation to CVD mortality and all-cause mortality. Hazard ratio (95% confidence interval) obtained from Cox proportional hazards models, using the lowest category as the reference. CVD, cardiovascular diseases; FLI, Fatty Liver Index. Model 2 adjusted for sex and age. Model 3, as model 2 and additionally adjusted for systolic blood pressure, statin use, smoking status, alcohol consumption, time since last myocardial infarction, and fasting.
(DOCX)

S5 Table HRs (95% CIs) for FLI in relation to CVD mortality and all-cause mortality excluding the first 2 y of follow-up in 4008 post-MI patients of the Alpha Omega Cohort. Hazard ratio (95% confidence interval) obtained from Cox proportional hazards models, using the lowest category as the reference. CVD, cardiovascular diseases; FLI, Fatty Liver Index. Model 2 adjusted for sex and age. Model 3, as model 2 and additionally adjusted for systolic blood pressure, statin use, smoking status, alcohol consumption, time since last myocardial infarction, and fasting.
(DOCX)

S6 Table HRs (95% CIs) for FLI in relation to CVD mortality and all-cause mortality using the first 10 years of follow-up in 4165 post-MI patients of the Alpha Omega Cohort. Hazard ratio (95% confidence interval) obtained from Cox proportional hazards models, using the lowest category as the reference. CVD, cardiovascular diseases; FLI, Fatty Liver Index. Model 2 adjusted for sex and age. Model 3, as model 2 and additionally adjusted for systolic blood pressure, statin use, smoking status, alcohol consumption, time since last myocardial infarction, and fasting.
(DOCX)

S7 Table Mean absolute change and relative change of FLI categories in 1678 post-MI patients of the Alpha Omega Cohort with repeated measurements after 40 months of follow-up. (DOCX)
