# Supplementary figures and images for: Fatty Liver Index and mortality after myocardial infarction: A prospective analysis in the Alpha Omega Cohort
Source: PLoS One. 2023 Sep 8;18(9):e0287467. doi: 10.1371/journal.pone.0287467 (PMC10490853; doi:10.1371/journal.pone.0287467)

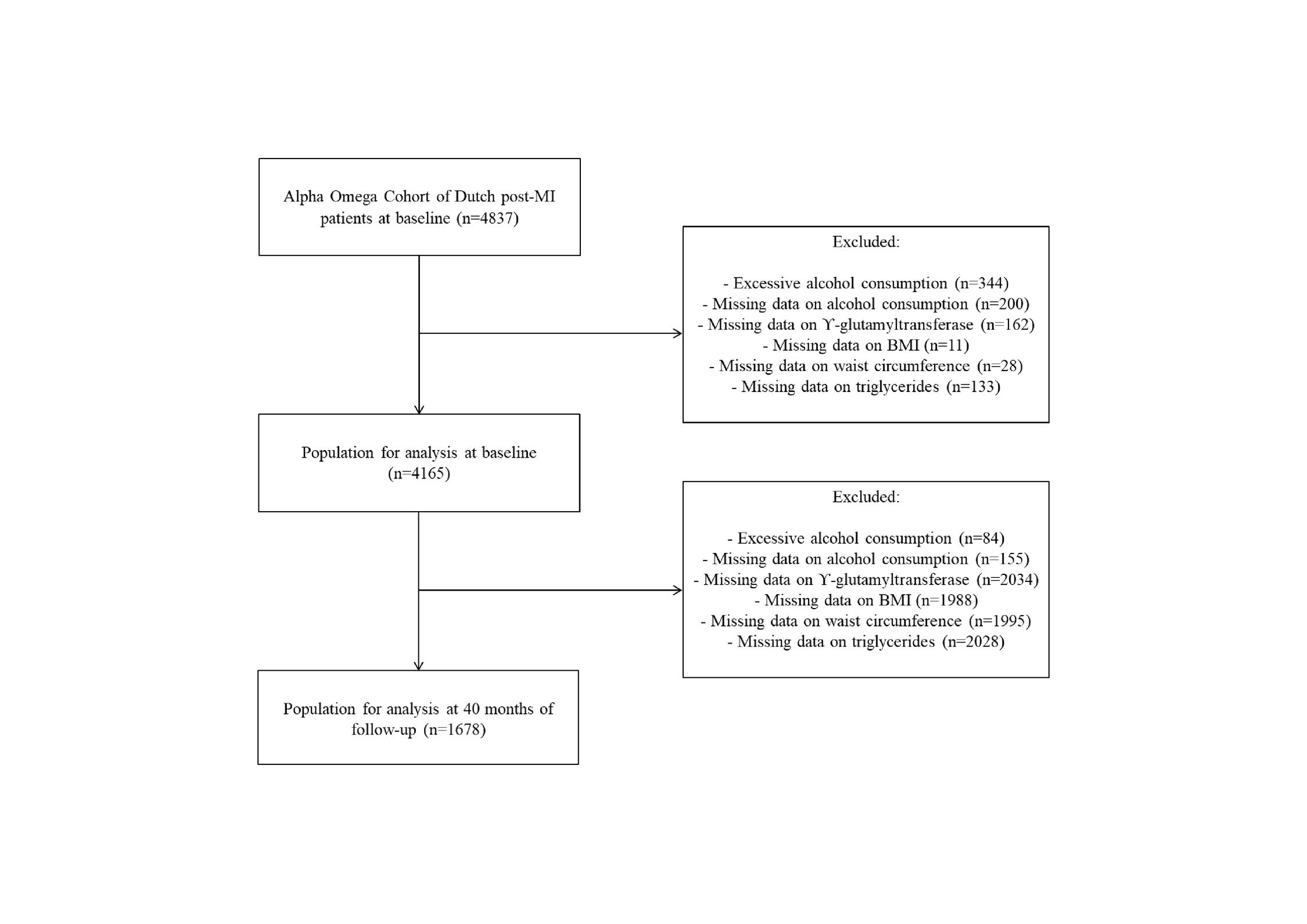

Supplement: S1 Fig — (TIF) [file pone.0287467.s001.tif]

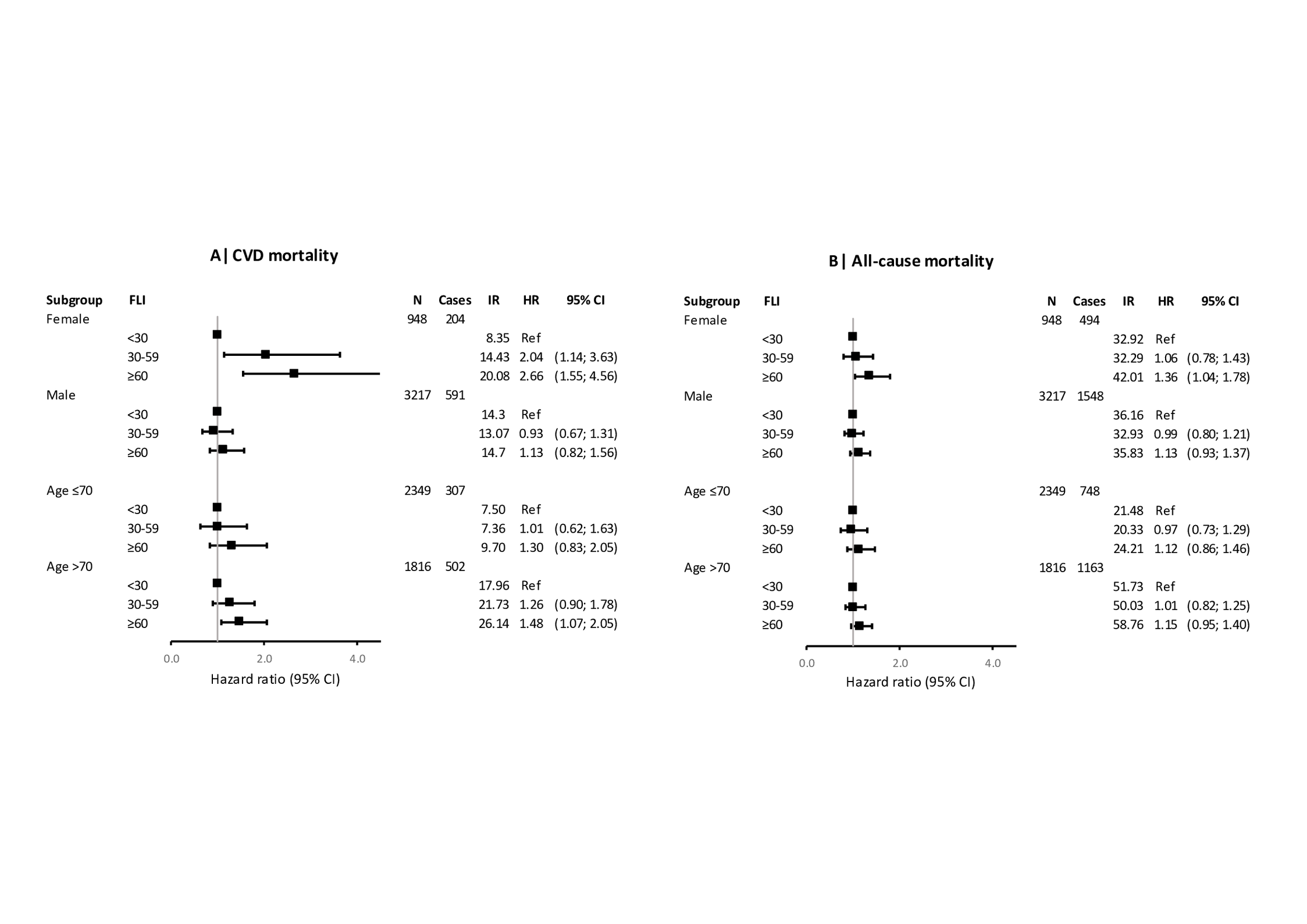

Supplement: S2 Fig — Hazard ratios (95% confidence interval) for FLI in relation to CVD mortality (A) and all-cause mortality (B), stratified for sex and age (≤70 y and >70 y) in 4165 post-MI patients of the Alpha Omega Cohort. Hazard ratios are adjusted for age (when stratified for sex), sex (when stratified for age), systolic blood pressure, smoking status, alcohol consumption, time since last myocardial infarction, and fasting. CVD, cardiovascular diseases; FLI, Fatty Liver Index; HR, Hazard ratio; 95% CI, 95% confidence interval; IR, Incidence rate (per 1000 person-years). (TIF) [file pone.0287467.s002.tif]

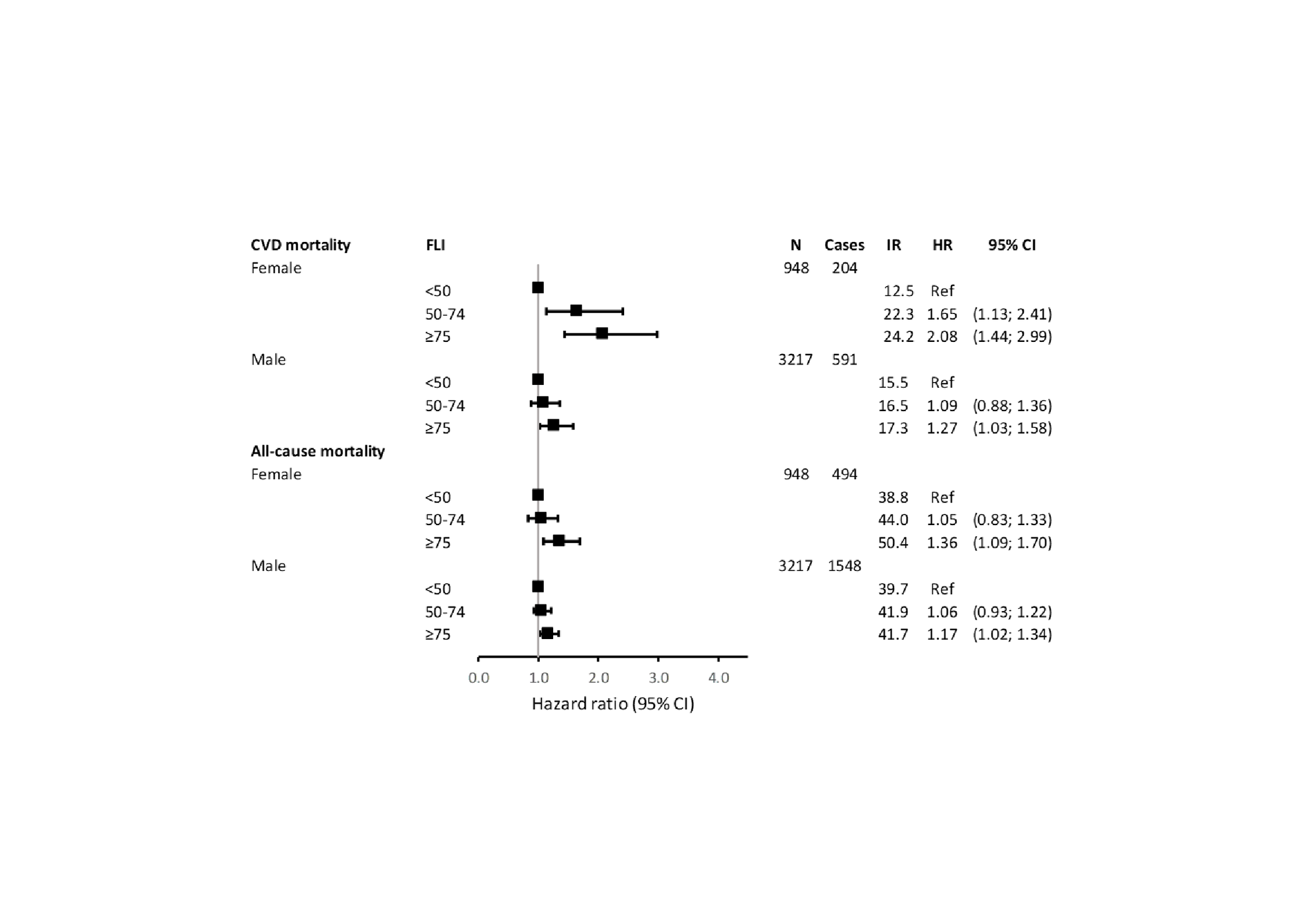

Supplement: S3 Fig — Hazard ratios (95% confidence interval) for tertiles of FLI in relation to CVD mortality (A) and all-cause mortality (B), stratified for sex in 4165 post-MI patients of the Alpha Omega Cohort. Hazard ratios are adjusted for age, systolic blood pressure, smoking status, alcohol consumption, time since last myocardial infarction, and fasting. CVD, cardiovascular diseases; FLI, Fatty Liver Index; HR, Hazard ratio; 95% CI, 95% confidence interval; IR, Incidence rate (per 1000 person-years). (TIF) [file pone.0287467.s003.tif]

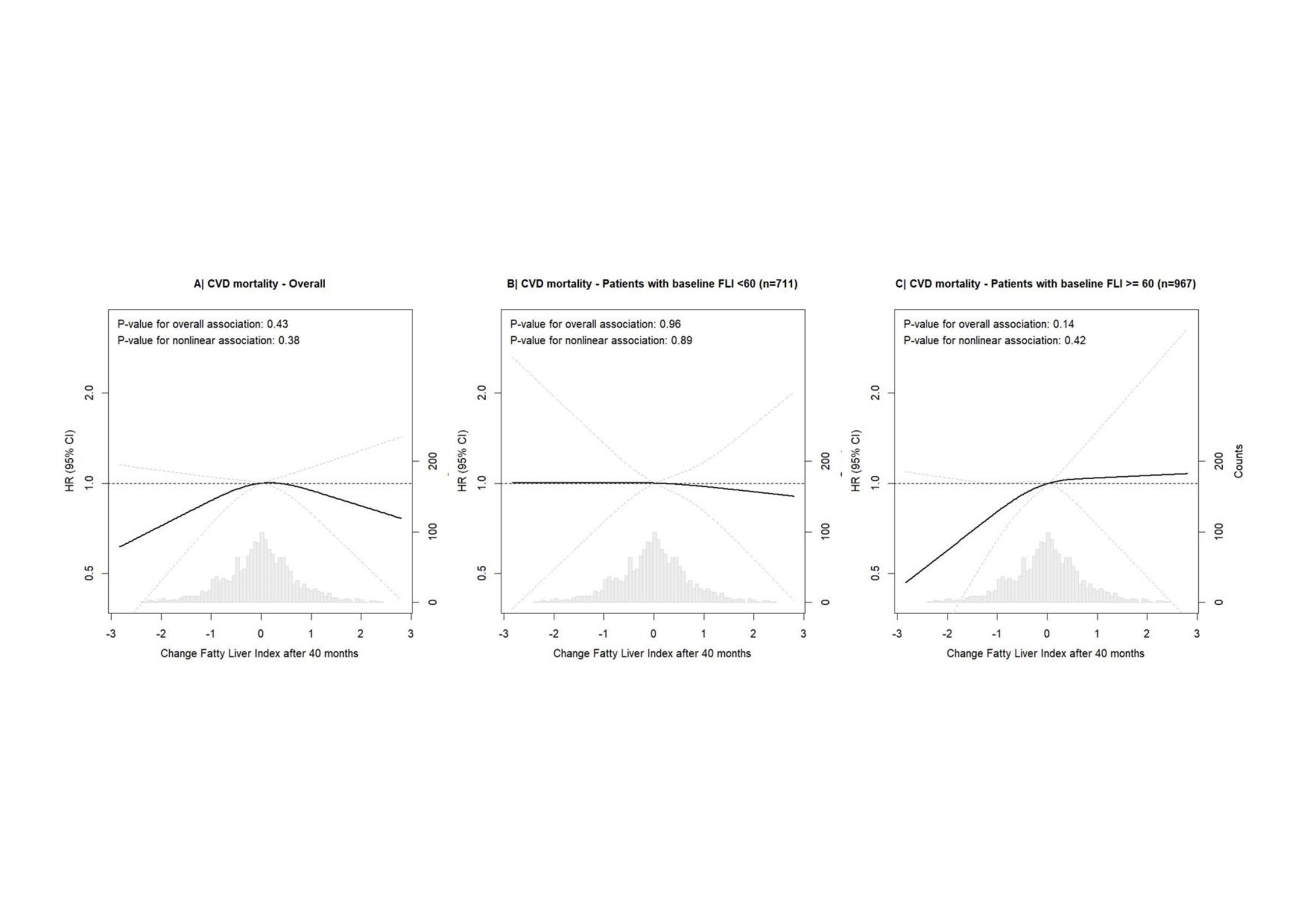

Supplement: S4 Fig — Associations in hazard ratios of change of FLI (expressed in Z-scores) in relation to CVD mortality in 1678 post-MI patients of the Alpha Omega Cohort, overall (A), for patients with baseline FLI <60 (B), and for patients with baseline FLI ≥60 (C). Hazard ratios with 95% CIs (dotted line) were modeled using restricted cubic splines. Three knots for FLI are located at the 10th, 50th, and 90th percentiles. Hazard ratios are adjusted for age, sex, systolic blood pressure, smoking status, alcohol consumption, time since last myocardial infarction, and fasting. CVD, cardiovascular diseases; FLI, Fatty Liver Index. (TIF) [file pone.0287467.s004.tif]

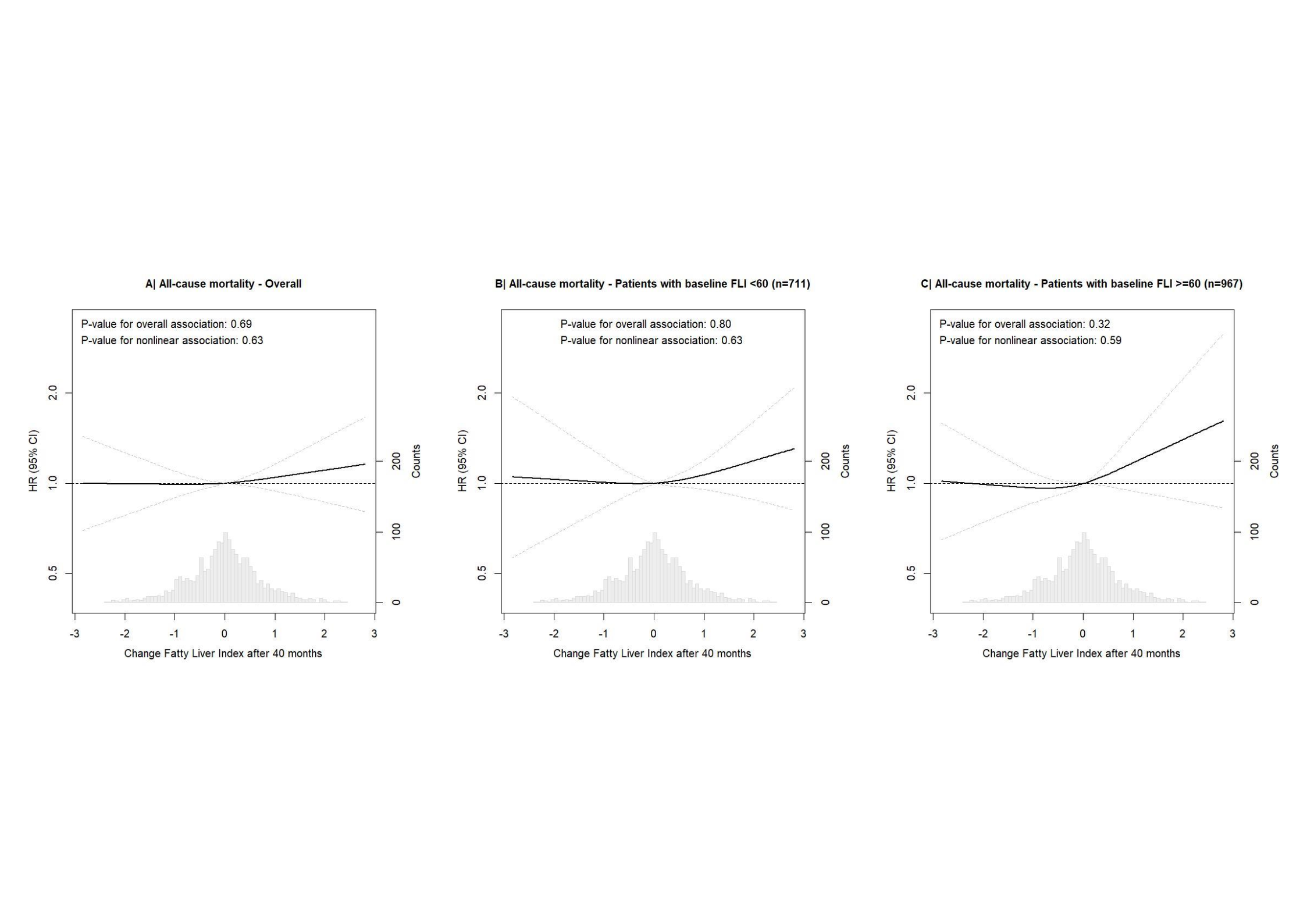

Supplement: S5 Fig — Associations in hazard ratios of change of FLI (expressed in Z-scores) in relation to CVD mortality in 1678 post-MI patients of the Alpha Omega Cohort, overall (A), for patients with baseline FLI <60 (B), and for patients with baseline FLI ≥60 (C). Hazard ratios with 95% CIs (dotted line) were modeled using restricted cubic splines. Three knots for FLI are located at the 10th, 50th, and 90th percentiles. Hazard ratios are adjusted for age, sex, systolic blood pressure, smoking status, alcohol consumption, time since last myocardial infarction, and fasting. FLI, Fatty Liver Index. (TIF) [file pone.0287467.s005.tif]
