# Supplementary material for: Fatty Liver Index and mortality after myocardial infarction: A prospective analysis in the Alpha Omega Cohort
Source: PLoS One. 2023 Sep 8;18(9):e0287467. doi: 10.1371/journal.pone.0287467 (PMC10490853; doi:10.1371/journal.pone.0287467)
Supplement: S1 Table — Hazard ratio (95% confidence interval) obtained from Cox proportional hazards models, using the lowest category as the reference. CVD, cardiovascular diseases; FLI, Fatty Liver Index. Model 2 adjusted for sex and age. Model 3, as model 2 and additionally adjusted for systolic blood pressure, statin use, smoking status, alcohol consumption, time since last myocardial infarction, and fasting. (DOCX) [file pone.0287467.s006.docx]

|  | Fatty Liver Index | | |
| --- | --- | --- | --- |
|  | <30 (n=384) | ≥30-<60 (n=1241) | ≥60 (n=1538) |
| CVD mortality |  |  |  |
| Cases | 58 | 218 | 309 |
| Person-years | 4210 | 14,133 | 16,677 |
| Incidence rate (per 1000 person-years) | 13.8 | 15.4 | 18.5 |
| Model 1 | 1.00 | 1.06 (0.80; 1.39) | 1.30 (0.99; 1.70) |
| Model 2 | 1.00 | 1.12 (0.85; 1.48) | 1.43 (1.09; 1.88) |
| Model 3 | 1.00 | 1.19 (0.89; 1.60) | 1.45 (1.09; 1.93) |
| All-cause mortality |  |  |  |
| Cases | 172 | 544 | 748 |
| Person-years | 4210 | 14,133 | 16,677 |
| Incidence rate (1000 per person-years) | 40.9 | 38.5 | 44.9 |
| Model 1 | 1.00 | 0.91 (0.77; 1.07) | 1.09 (0.93; 1.28) |
| Model 2 | 1.00 | 0.95 (0.80; 1.12) | 1.16 (0.99; 1.37) |
| Model 3 | 1.00 | 0.99 (0.84; 1.18) | 1.16 (0.98; 1.38) |
