# Supplementary material for: Fatty Liver Index and mortality after myocardial infarction: A prospective analysis in the Alpha Omega Cohort
Source: PLoS One. 2023 Sep 8;18(9):e0287467. doi: 10.1371/journal.pone.0287467 (PMC10490853; doi:10.1371/journal.pone.0287467)
Supplement: S2 Table — Hazard ratio (95% confidence interval) obtained from Cox proportional hazards models, using the lowest category as the reference. CVD, cardiovascular diseases; FLI, Fatty Liver Index. Model 2 adjusted for sex and age. Model 3, as model 2 and additionally adjusted for systolic blood pressure, statin use, smoking status, alcohol consumption, time since last myocardial infarction, and fasting. (DOCX) [file pone.0287467.s007.docx]

|  | Fatty Liver Index | | |
| --- | --- | --- | --- |
|  | <30 (n=331) | ≥30-<60 (n=1100) | ≥60 (n=1877) |
| CVD mortality |  |  |  |
| Cases | 47 | 197 | 353 |
| Person-years | 3696 | 12,646 | 20,897 |
| Incidence rate (per 1000 person-years) | 12.7 | 15.6 | 16.9 |
| Model 1 | 1.00 | 1.13 (0.84; 1.53) | 1.26 (0.95; 1.68) |
| Model 2 | 1.00 | 1.17 (0.86; 1.58) | 1.39 (1.06; 1.86) |
| Model 3 | 1.00 | 1.28 (0.94; 1.74) | 1.44 (1.08; 1.93) |
| All-cause mortality |  |  |  |
| Cases | 136 | 472 | 859 |
| Person-years | 3696 | 12,646 | 20,897 |
| Incidence rate (per 1000 person-years) | 36.8 | 37.3 | 41.1 |
| Model 1 | 1.00 | 0.97 (0.80; 1.17) | 1.09 (0.92; 1.30) |
| Model 2 | 1.00 | 1.00 (0.83; 1.20) | 1.19 (1.00; 1.42) |
| Model 3 | 1.00 | 1.10 (0.91; 1.32) | 1.25 (1.05; 1.49) |
