# Supplementary material for: Fatty Liver Index and mortality after myocardial infarction: A prospective analysis in the Alpha Omega Cohort
Source: PLoS One. 2023 Sep 8;18(9):e0287467. doi: 10.1371/journal.pone.0287467 (PMC10490853; doi:10.1371/journal.pone.0287467)
Supplement: S3 Table — Hazard ratio (95% confidence interval) obtained from Cox proportional hazards models, using the lowest category as the reference. CVD, cardiovascular diseases; FLI, Fatty Liver Index. Model 2 adjusted for sex and age. Model 3, as model 2 and additionally adjusted for systolic blood pressure, statin use, smoking status, alcohol consumption, time since last myocardial infarction, and fasting. a Log transformed due to skewness of the data. b Fasting and non-fasting triglycerides. c Missing data for 48 patients (n = 4117). (DOCX) [file pone.0287467.s008.docx]

|  | CVD mortality | All-cause mortality |
| --- | --- | --- |
| BMI, kg/m^2^ |  |  |
| Cases | 795 | 1934 |
| Person-years | 46,072 | 46,072 |
| Incidence rate (per 1000 person-years) | 17.3 | 41.9 |
| Model 1 | 1.05 (0.98; 1.12) | 0.99 (0.94; 1.03) |
| Model 2 | 1.11 (1.03; 1.20) | 1.05 (1.00; 1.10) |
| Model 3 | 1.12 (1.05; 1.21) | 1.07 (1.02; 1.12) |
| Waist circumference, cm |  |  |
| Cases | 795 | 1934 |
| Person-years | 46,072 | 46,072 |
| Incidence rate (per 1000 person-years) | 17.3 | 41.9 |
| Model 1 | 1.12 (1.04; 1.20) | 1.09 (1.05; 1.15) |
| Model 2 | 1.17 (1.09; 1.26) | 1.13 (1.08; 1.19) |
| Model 3 | 1.18 (1.10; 1.26) | 1.14 (1.09; 1.20) |
| Gamma-glutamyltransferase^a^, U/L |  |  |
| Cases | 795 | 1934 |
| Person-years | 46,072 | 46,072 |
| Incidence rate (per 1000 person-years) | 17.3 | 41.9 |
| Model 1 | 1.13 (1.08; 1.18) | 1.18 (1.09; 1.27) |
| Model 2 | 1.19 (1.14; 1.24) | 1.16 (1.12; 1.20) |
| Model 3 | 1.20 (1.15; 1.26) | 1.16 (1.13; 1.20) |
| Triglycerides^a,b^, mmol/L |  |  |
| Cases | 795 | 1934 |
| Person-years | 46,072 | 46,072 |
| Incidence rate (per 1000 person-years) | 17.3 | 41.9 |
| Model 1 | 1.06 (1.00; 1.13) | 1.04 (1.00; 1.09) |
| Model 2 | 1.11 (1.04; 1.19) | 1.08 (1.04; 1.13) |
| Model 3 | 1.07 (1.00; 1.15) | 1.05 (1.00; 1.10) |
| AST/ALT ratio^c^ |  |  |
| Cases | 776 | 1894 |
| Person-years | 45,719 | 45,719 |
| Incidence rate (per 1000 person-years) | 17.0 | 41.4 |
| Model 1 | 1.08 (1.05; 1.11) | 1.08 (1.05; 1.10) |
| Model 2 | 1.05 (1.01; 1.10) | 1.05 (1.02; 1.08) |
| Model 3 | 1.05 (1.00; 1.09) | 1.05 (1.02; 1.08) |
