# Supplementary material for: Fatty Liver Index and mortality after myocardial infarction: A prospective analysis in the Alpha Omega Cohort
Source: PLoS One. 2023 Sep 8;18(9):e0287467. doi: 10.1371/journal.pone.0287467 (PMC10490853; doi:10.1371/journal.pone.0287467)
Supplement: S4 Table — Hazard ratio (95% confidence interval) obtained from Cox proportional hazards models, using the lowest category as the reference. CVD, cardiovascular diseases; FLI, Fatty Liver Index. Model 2 adjusted for sex and age. Model 3, as model 2 and additionally adjusted for systolic blood pressure, statin use, smoking status, alcohol consumption, time since last myocardial infarction, and fasting. (DOCX) [file pone.0287467.s009.docx]

|  | Fatty Liver Index | |
| --- | --- | --- |
|  | <60 (n=1646) | ≥60 (n=2519) |
| CVD mortality |  |  |
| Cases | 284 | 511 |
| Person-years | 19,211 | 28,644 |
| Incidence rate (per 1000 person-years) | 14.8 | 17.8 |
| Model 1 | 1.00 | 1.22 (1.06; 1.40) |
| Model 2 | 1.00 | 1.33 (1.16; 1.53) |
| Model 3 | 1.00 | 1.31 (1.13; 1.50) |
| All-cause mortality |  |  |
| Cases | 729 | 1275 |
| Person-years | 19,211 | 28,644 |
| Incidence rate (per 1000 person-years) | 37.9 | 44.5 |
| Model 1 | 1.00 | 1.12 (1.03; 1.23) |
| Model 2 | 1.00 | 1.22 (1.11; 1.33) |
| Model 3 | 1.00 | 1.18 (1.08; 1.30) |
