# Supplementary material for: Fatty Liver Index and mortality after myocardial infarction: A prospective analysis in the Alpha Omega Cohort
Source: PLoS One. 2023 Sep 8;18(9):e0287467. doi: 10.1371/journal.pone.0287467 (PMC10490853; doi:10.1371/journal.pone.0287467)
Supplement: S5 Table — Hazard ratio (95% confidence interval) obtained from Cox proportional hazards models, using the lowest category as the reference. CVD, cardiovascular diseases; FLI, Fatty Liver Index. Model 2 adjusted for sex and age. Model 3, as model 2 and additionally adjusted for systolic blood pressure, statin use, smoking status, alcohol consumption, time since last myocardial infarction, and fasting. (DOCX) [file pone.0287467.s010.docx]

|  | Fatty Liver Index | | |
| --- | --- | --- | --- |
|  | <30 (n=375) | ≥30-<60 (n=1219) | ≥60 (n=2414) |
| CVD mortality |  |  |  |
| Cases | 54 | 203 | 458 |
| Person-years | 3451 | 11,853 | 22,572 |
| Incidence rate (per 1000 person-years) | 15.6 | 17.1 | 20.3 |
| Model 1 | 1.00 | 1.05 (0.78; 1.39) | 1.25 (0.95; 1.64) |
| Model 2 | 1.00 | 1.10 (0.82; 1.64) | 1.43 (1.09; 1.87) |
| Model 3 | 1.00 | 1.22 (0.89; 1.67) | 1.48 (1.10; 2.00) |
| All-cause mortality |  |  |  |
| Cases | 163 | 514 | 1100 |
| Person-years | 3451 | 11,853 | 22,572 |
| Incidence rate (per 1000 person-years) | 47.2 | 43.4 | 48.7 |
| Model 1 | 1.00 | 0.89 (0.75; 1.06) | 1.02 (0.75; 1.06) |
| Model 2 | 1.00 | 0.92 (0.77; 1.09) | 1.13 (0.97; 1.33) |
| Model 3 | 1.00 | 0.99 (0.83; 1.17) | 1.17 (0.99; 1.37) |
