# Supplementary material for: Fatty Liver Index and mortality after myocardial infarction: A prospective analysis in the Alpha Omega Cohort
Source: PLoS One. 2023 Sep 8;18(9):e0287467. doi: 10.1371/journal.pone.0287467 (PMC10490853; doi:10.1371/journal.pone.0287467)
Supplement: S6 Table — Hazard ratio (95% confidence interval) obtained from Cox proportional hazards models, using the lowest category as the reference. CVD, cardiovascular diseases; FLI, Fatty Liver Index. Model 2 adjusted for sex and age. Model 3, as model 2 and additionally adjusted for systolic blood pressure, statin use, smoking status, alcohol consumption, time since last myocardial infarction, and fasting. (DOCX) [file pone.0287467.s011.docx]

|  | Fatty Liver Index | | |
| --- | --- | --- | --- |
|  | <30 (n=384) | ≥30-<60 (n=1262) | ≥60 (n=2519) |
| CVD mortality |  |  |  |
| Cases | 39 | 137 | 328 |
| Person-years | 3350 | 11,170 | 21,718 |
| Incidence rate (per 1000 person-years) | 11.6 | 12.3 | 15.1 |
| Model 1 | 1.00 | 1.05 (0.73; 1.49) | 1.29 (0.93; 1.80) |
| Model 2 | 1.00 | 1.06 (0.75; 1.52) | 1.41 (1.01; 1.96) |
| Model 3 | 1.00 | 1.11 (0.80; 1.68) | 1.39 (0.99; 1.97) |
| All-cause mortality |  |  |  |
| Cases | 120 | 342 | 781 |
| Person-years | 3350 | 11,170 | 21,718 |
| Incidence rate (per 1000 person-years) | 35.8 | 30.6 | 36.0 |
| Model 1 | 1.00 | 0.85 (0.69; 1.05) | 1.00 (0.83; 1.22) |
| Model 2 | 1.00 | 0.86 (0.70; 1.06) | 1.08 (0.89; 1.30) |
| Model 3 | 1.00 | 0.93 (0.75; 1.15) | 1.06 (0.87; 1.30) |
