# Supplementary material for: Fatty Liver Index and mortality after myocardial infarction: A prospective analysis in the Alpha Omega Cohort
Source: PLoS One. 2023 Sep 8;18(9):e0287467. doi: 10.1371/journal.pone.0287467 (PMC10490853; doi:10.1371/journal.pone.0287467)
Supplement: S7 Table — (DOCX) [file pone.0287467.s012.docx]

| Mean absolute change | |
| --- | --- |
| FLI | 10.5 ± 9.3 |
| BMI | 1.3 ± 1.1 |
| Waist circumference | 4.5 ± 4.0 |
| Triglycerides | 0.63 ± 0.69 |
| Gamma-glutamyltransferase | 15.1 ± 41.8 |
| Relative change FLI categories, n (%) | |
| <30 to 30-<60 | 46 (3) |
| <30 to ≥60 | 6 (0.4) |
| 30-<60 to ≥60 | 136 (8) |
| ≥60 to 30-<60 | 168 (10) |
| ≥60 to <30 | 9 (1) |
| 30-<60 to <30 | 89 (5) |
